# Coronary flow velocity reserve by echocardiography: feasibility, reproducibility and agreement with PET in overweight and obese patients with stable and revascularized coronary artery disease

**DOI:** 10.1186/s12947-016-0066-3

**Published:** 2016-06-07

**Authors:** Rasmus Huan Olsen, Lene Rørholm Pedersen, Martin Snoer, Thomas Emil Christensen, Adam Ali Ghotbi, Philip Hasbak, Andreas Kjaer, Steen B. Haugaard, Eva Prescott

**Affiliations:** 1 Department of Cardiology, Bispebjerg Hospital, University of Copenhagen, Building 67, Bispebjerg Bakke 23, DK-2400 Copenhagen NV, Denmark; 2 Department of Clinical Physiology, Nuclear Medicine & PET, Rigshospitalet, University of Copenhagen, Copenhagen, Denmark; 3 Department of Internal Medicine and Clinical Research Centre, Amager and Hvidovre Hospitals, University of Copenhagen, Copenhagen, Denmark

**Keywords:** Coronary artery disease, Coronary flow reserve, Echocardiography, Microvascular function, Obesity, Positron emission tomography

## Abstract

**Background:**

Coronary flow velocity reserve (CFVR) measured by transthoracic Doppler echocardiography of the LAD is used to assess microvascular function but validation studies in clinical settings are lacking. We aimed to assess feasibility, reproducibility and agreement with myocardial flow reserve (MFR) measured by PET in overweight and obese patients.

**Methods:**

Participants with revascularized coronary artery disease were examined by CFVR. Subgroups were examined by repeated CFVR (reproducibility) or Rubidium-82-PET (agreement). To account for time variation, results were computed for scans performed within a week (1-week) and for all scans regardless of time gap (total) and to account for scar tissue for patients with and without previous myocardial infarction (MI).

**Results:**

Eighty-six patients with median BMI 30.9 (IQR 29.4–32.9) kg × m^−2^ and CFVR 2.29 (1.90–2.63) were included. CFVR was feasible in 83 (97 %) using a contrast agent in 14 %. For reproducibility overall (*n* = 21) limits of agreement (LOA) were (−0.75;0.71), within-subjects coefficient of variation (CV) 11 %, and reliability 0.84. For reproducibility within 1-week (*n* = 13) LOA were (−0.33;0.25), within-subjects CV 5 %, and reliability 0.97. Agreement with MFR of the LAD territory (*n* = 35) was without significant bias and overall LOA were (−1.40;1.46). Agreement was best for examinations performed within 1-week of participants without MI of the LAD-territory (*n* = 12); LOA = (−0.68;0.88).

**Conclusions:**

CFVR was highly feasible with a good reproducibility on par with other contemporary measures applied in cardiology. Agreement with MFR was acceptable, though discrepancy related to prior MI has to be considered. CFVR of LAD is a valid tool in overweight and obese patients.

## Background

Coronary flow reserve (CFR) is an integrated measure of coronary macro- and microvascular morphology and function and is defined as the ratio of hyperaemic coronary blood flow during maximum vasodilation of the coronary vascular bed to resting coronary blood flow [1–3]. In absence of significant coronary artery stenosis, CFR is considered a quantitative measure of coronary microvascular function [2]. Coronary microvascular function is increasingly being recognised as an important pathophysiologic and prognostic factor in cardiovascular disorders [2, 4]. Microvascular function is reduced in coronary artery disease (CAD), even in territories without prior coronary artery stenosis [5], and impaired microvascular function carries a poor prognosis [4, 6].

Coronary flow velocity reserve (CFVR) measured by transthoracic echocardiography (TTE) with spectral Doppler measurement of coronary artery flow velocity (CFV) is a non-invasive, non-ionising method and the least expensive for measurement of coronary microvascular function. CFVR has both diagnostic and prognostic implications and may be a useful translational tool for risk-stratification and to evaluate potential effects of intervention both in preclinical and clinical proof-of-concept studies [3, 6–10].

The left anterior descending coronary artery (LAD) is the more approachable coronary artery for measurement of CFVR [11]. Agreement with intracoronary Doppler flow wire has been established in small studies of unselected patients referred for coronary angiography (CAG) [12–15].

In a European cohort, 82 % of the patients diagnosed with coronary artery disease (CAD) were overweight or obese [16], and two thirds of the US population are estimated to be overweight or obese [17]. Despite this, CFVR by TTE Doppler of LAD has previously only been validated against myocardial flow reserve (MFR) by positron emission tomography (PET), the non-invasive gold standard method of myocardial perfusion, in ten healthy, young, male subjects [18]. Thus with reference to the obesity epidemic and an increasing interest in CFR as a measure of coronary microvascular function in CAD [2, 4], validation of CFVR in overweight and obese patients is needed.

The aim of the study was therefore to validate CFVR by TTE Doppler of the LAD in an overweight and obese cohort of stable and revascularized CAD patients in terms of:

1. Assessing feasibility,
2. Estimating reproducibility, and
3. Estimating agreement with MFR measured by PET.

## Methods

### Study participants

A total of 86 participants were recruited at the Department of Cardiology, Bispebjerg University Hospital among stable patients awaiting cardiac rehabilitation (*n* = 16) and patients enrolled in the CUT-IT trial (*n* = 70), a study of stable, overweight CAD patients [19]. All participants had been revascularized according to guidelines and had left ventricular ejection fraction above 35 %. Patients were invited to participate in the feasibility study and sub-studies without the investigators having prior knowledge of the echocardiographic feasibility and quality. All patients were included in the feasibility study (*n* = 86).

For the study of CFVR reproducibility, 31 participants were randomly invited, seven declined (agreed to participate in the feasibility study only), measurement was not feasible in three; thus 21 participants were examined twice by the same experienced echocardiographer.

**Fig. 1:**
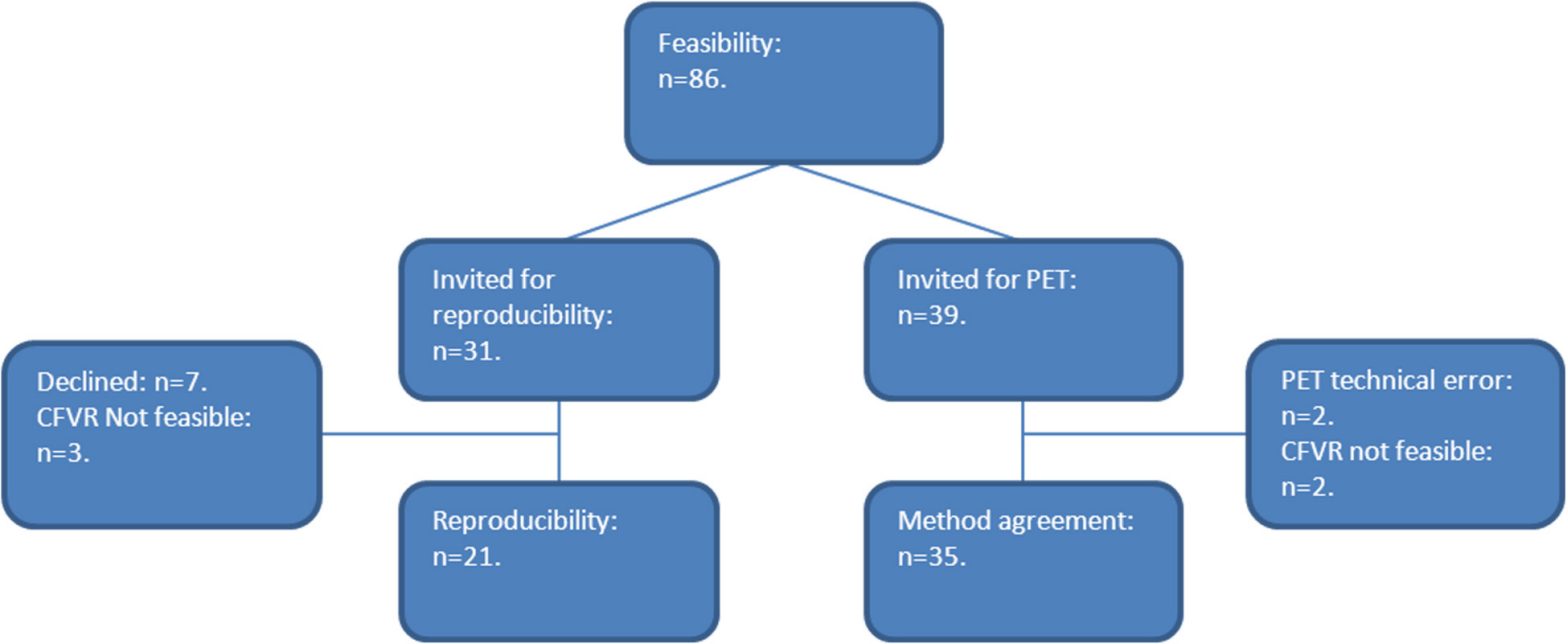
Overview of participants in the different parts of the study

For the study on agreement with MFR, thirty-nine participants underwent PET imaging; of them two were excluded from the analysis due to technical errors (specifically protracted infusions resulting in too little tracer infusion or mistiming of infusion and data acquisition) resulting in too few counts during the stress part of the examination, and in two CFVR was not feasible. Thus, agreement between TTE and PET could be evaluated in a total of 35 participants. Examinations were performed at least 6 months after latest myocardial infarct (MI), Percutaneous Coronary Intervention (PCI) or Coronary Artery Bypass Graft (CABG). Information about MI, PCI and CABG were obtained from the medical records. An overview of the participants in the substudies is given in Fig. 1.

The study complies with the Declaration of Helsinki and was approved by the Research Ethics Committee for the Capital Region of Denmark (H-1-2009-127 and H-4-2010-146). All participants gave informed written consent.

### Coronary flow velocity reserve - CFVR

CFVR was measured by two experienced echocardiographers (RHO, MS) using a high-frequency broadband transducer, either S6 with Vivid E9 (GE Healthcare, Horten, Norway) or S8 with iE33 (Philips Medical Systems, Andover, MA, USA), with second harmonics as previously described [8, 20]. All patients were instructed to abstain from caffeine for 24 h before the examination. With the patient in the left lateral decubitus position LAD was visualized by colour Doppler along the anterior interventricular sulcus as distal as possible with flow towards the transducer; distal (cross-sectional view of the apex by a modified, craniomedially displaced foreshortened apical 5- or 2-chamber view) or alternatively mid-distal (modified low short-axis view) [21, 22]. Position, angle and rotation of the probe were then optimized for measuring the characteristic, prevalent diastolic component of the biphasic flow in the LAD. CFV was measured as the peak diastolic flow using a pulsed-wave (PW) Doppler sample volume of 3–4 mm, at rest and during myocardial hyperaemia induced by intravenous infusion 140 μg × kg^−1^ × min^−1^of adenosine (2 min) or dipyridamole (6 min). This dosage of coronary vasodilators have been shown to give similar responses in CFV [23].

Before and during hyperaemia care was taken to ensure that measurements were done at the same angle on the same segment of the LAD. If CFV was unobtainable or the quality of the CFV envelope was considered inadequate or incomplete, an intravenous contrast agent, sulphurhexafluorid (SonoVue, Bracco Imaging Skandinavia AB, Hisings Backa, Sweden) or perflutren (Optison, GE Healthcare A/S, Brøndby, Denmark), was applied to enhance both the visualization of the colour Doppler delineation of the LAD and the outer edge of the CFV PW Doppler tracing. CFVR was calculated as the ratio between the highest CFV obtained during or after infusion and resting CFV using a mean of three consecutive cardiac cycles (6 to 10 if atrial fibrillation was present). Analyses were done offline by an investigator blinded to the other examinations.

**Fig. 2:**
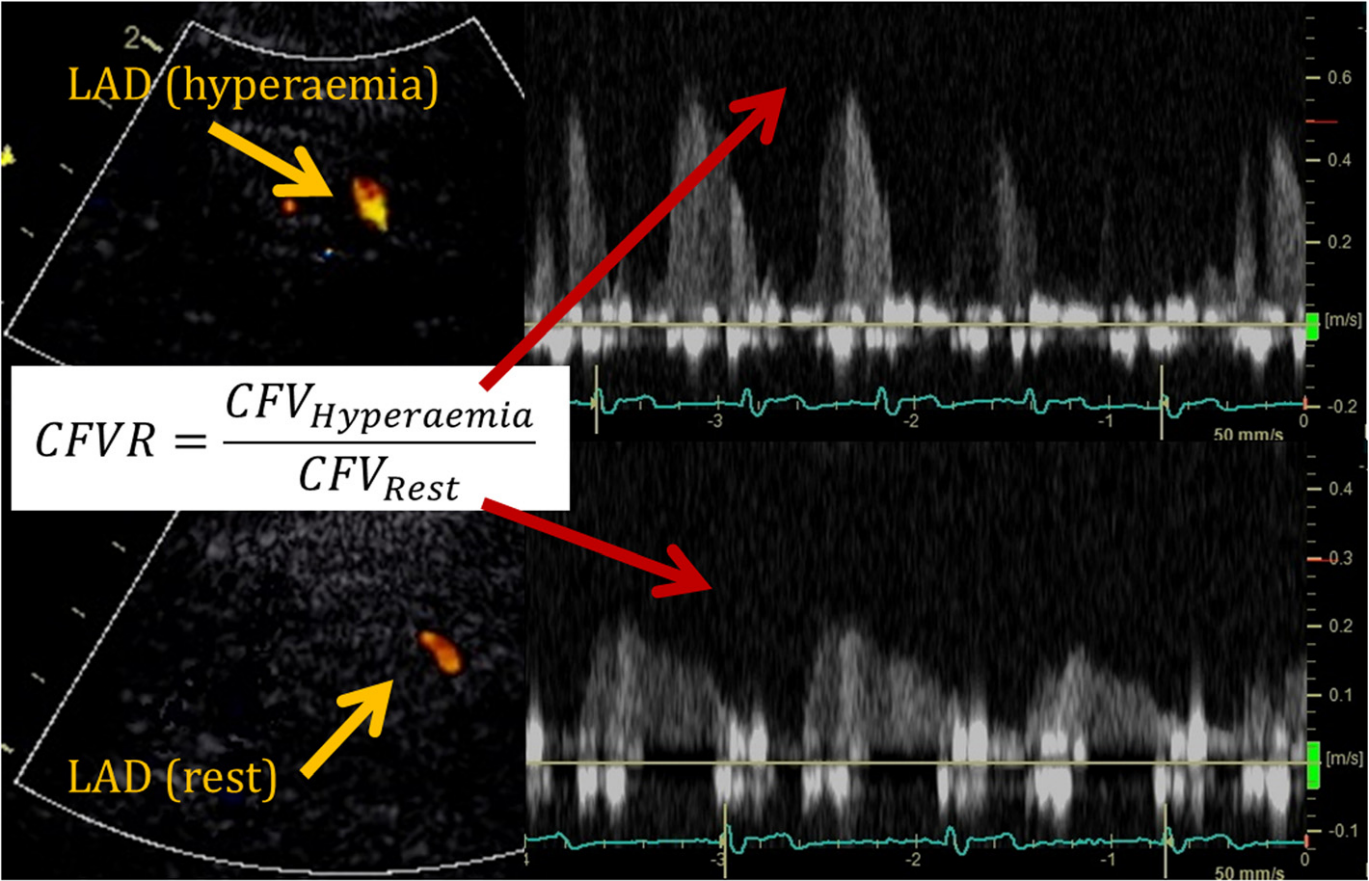
Example of CFVR measurement by Doppler TTE. Lower pictures show measurement at rest. Upper pictures show measurement during hyperaemia. Pictures to the left show the colour Doppler visualisation of the LAD. Pictures to the right show the pulsed wave Doppler measurement of the diastolic peak coronary flow velocity (CFV). Coronary flow velocity reserve (CFVR) of this participant was 2.86 (corresponding MFR_LAD_ was 2.57)

**Fig. 3:**
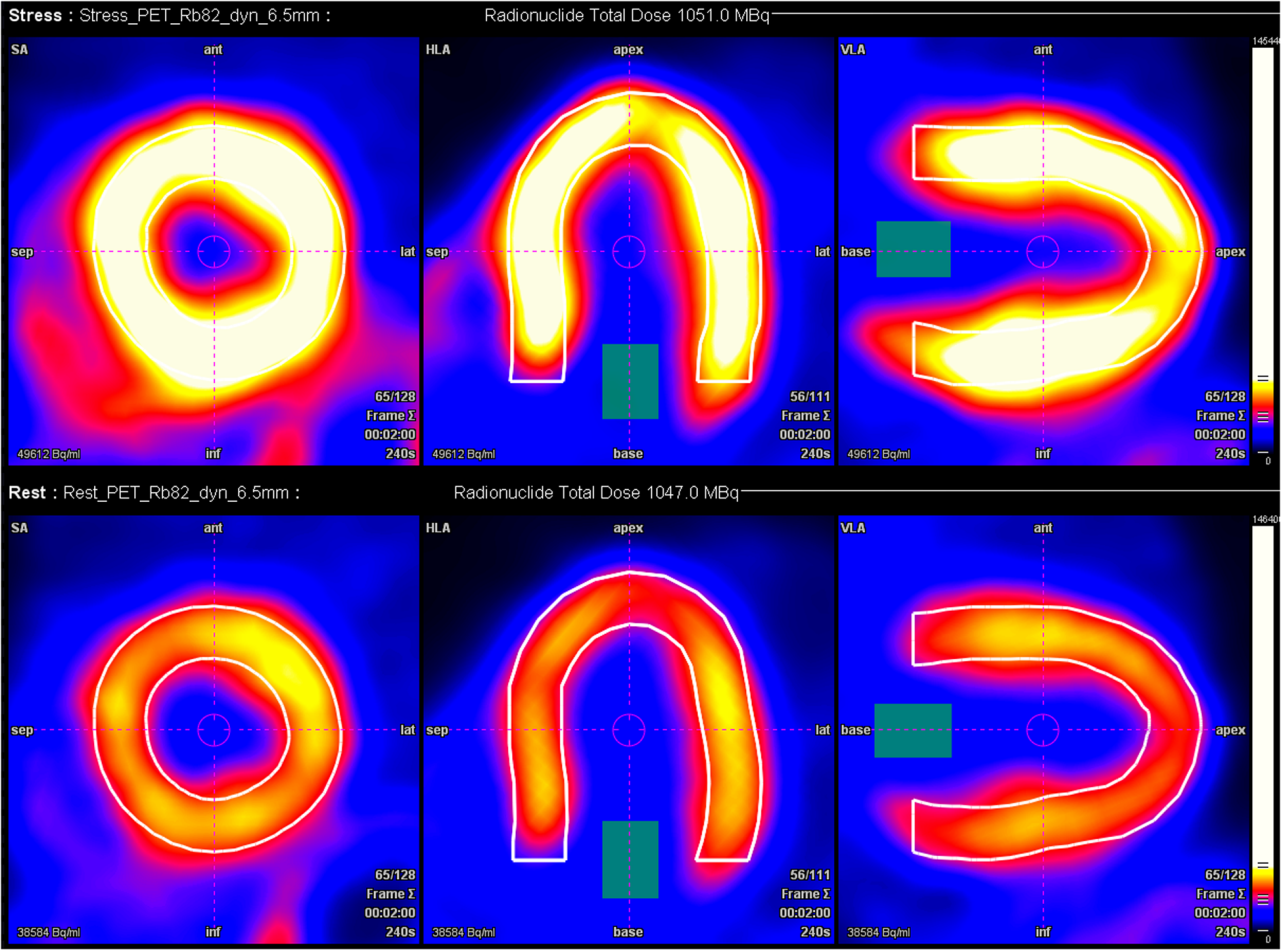
Example of MFR measurement by ^82^Rb PET. Lower row illustrates ^82^Rb-myocardial uptake at rest. Upper row illustrates ^82^Rb-myocardial uptake during hyperaemia. MFR_LAD_ of this participant was 2.57

We have previously reported inter and intra-observer variability of repeated off-line CFR readings with within-subject coefficient of variation (CV) and limits of agreement (LOA) of 5.5 % and ±0.21 (*n* = 39), and 7.5 % and ±0.29 (*n* = 10), respectively [24]. An example of CFVR and MFR measurement is shown in Figs. 2 and 3, respectively.

### Myocardial flow reserve - MFR

The PET scan was performed on Siemens Biograph mCT/PET 128 slice scanner (Siemens Healthcare, Erlangen, Germany). Patients underwent serial rest followed by stress imaging with Rubidium-82(^82^Rb) from a CardioGen82 ^82^Sr/^82^Rb generator (Bracco Diagnostics Inc., Princeton, NJ, USA). Our standard clinical protocol was used: An X-ray scout view over the chest was performed for positioning followed by low-dose computed tomography (CT) (120 kV, quality reference effective mAs = 11, rotation 0.5 s, pitch 1.5, collimation 16 × 1.2 mm) for attenuation correction of the rest emission data.^82^Rb was infused intravenously at a flow rate of 50 mL × min^−1^ and list mode 3D data acquisition was started with the tracer infusion continuing for 7 min. Adenosine was infused as stated above. Intravenous ^82^Rb infusion and list mode acquisition began 2.5 min after the start of adenosine infusion following the same protocol as for rest. Registration between PET and CT images was checked for evidence of patient motion and manual adjustments were made before reconstruction to correct for any minor motion. In cases of significant patient motion between PET and CT, an additional low-dose CT could be acquired at the end of the study. Both rest and stress dynamic images used for myocardial blood flow (MBF) quantification were reconstructed into 18 time frames (1 × 10, 8 × 5, 3 × 10, 2 × 20, 4 × 60 s) on a 128 × 128 matrix and 2 × zoom (voxel dimensions 3.18 × 3.18 × 2.03 mm) using 3D OSEM reconstruction (2 iterations, 21 subsets) with Siemens UltraHD-PET, a 6.5 mm Gaussian postfilter, attenuation and scatter corrected (including prompt gamma correction [25]). MBF quantification was performed using Syngo MBF software (Siemens Healthcare, Erlangen, Germany) based on a single-compartment model for ^82^Rb tracer kinetics [26]. Administered ^82^Rb-activity was 1110 MBq during rest and stress. Thus, the radiation dose for each patient was estimated to be 2.6 mSv in total for rest and stress [27]. MFR was calculated as: MBF during stress (mL × min^−1^ × g^−1^)/MBF during rest (mL × min^−1^ × g^−1^), both globally for the left ventricle (MFR_global_) and for the LAD territory (MFR_LAD_) with reference to the AHA 17 segment model [28].

### Reproducibility of CFVR and agreement with MFR

To avoid spillover effects, infusion of adenosine/dipyridamole was repeated with at least 24/72 h’ delay, respectively. In an attempt to minimize influence of fluctuations in CFR over time on measurement variability, we aimed at performing repeated exams within seven days. Due to logistics, including a world-wide pause in CardioGen82 supply, this was only possible in 13 (62 %) and 20 (57 %) of participants in the reproducibility and method agreement (PET) sub-studies, respectively. Participants agreed to avoid changes in medication during the study period. Regional perfusion and thus MFR_global_ was a priori assumed to be affected by presence of scar-tissue from previous MI [29]. Results are therefore given for the entire population, stratified by time between examinations with cut-off at one week and stratified by previous MI. For comparison with MFR_LAD_, only participants with previous MI of the LAD perfusion territory (MI_LAD_) were included in the MI subgroup. For comparison with MFR_global_, participants with any previous MI were included in the MI subgroup.

We explored whether correction for Rate-pressure product (RPP) would improve reproducibility of CFVR and agreement between MFR and CFVR. RPP, systolic blood pressure multiplied by heart rate, is a surrogate for myocardial work [29]. Under normal circumstances myocardial blood flow is determined by the work (O_2_ demand) of the heart via autoregulation. The autoregulation of myocardial perfusion is uncoupled during pharmacologically induced vasodilatory hyperaemia and the correlation between RPP and perfusion is reduced. In PET-studies, resting flow is often corrected for resting RPP and in some studies the usage is expanded to the calculation of a supplemental RPP corrected MFR [30]. Thus, a RPP corrected MFR and CFVR was calculated by multiplying MFR or CFVR by resting RPP and dividing by 10,000.

### Statistics

Unless stated otherwise, values are expressed as median (interquatile range [IQR]) for continuous variables and as count (%) for categorical variables. Continuous variables were evaluated by *t*-test or in case of non-normal distribution Kruskal-Wallis equality-of-populations rank test. Normal distribution was assessed graphically and by Shapiro-Wilk test for normality. Equality of variances was tested by Levenes test or in case of non-normal distribution Kolmogorov-Smirnov equality-of-distributions test. Categorical variables were evaluated using *χ* ^2^ or Fishers exact test if an expected cell frequency was <5. *P* < 0.05 in two-sided tests were considered statistically significant. Confidence intervals (CI) refer to 95 % confidence intervals. “Limits of agreement” (LOA) corresponds to the 95 % prediction interval of differences and was estimated for repeated examinations of CFVR and for the comparison of CFVR and MFR, and presented as mean difference (bias) and two times the standard deviation of the differences (2SD) [31, 32]. Coefficient of variation (CV) was calculated as the SD divided by the population mean.

Reliability relates the magnitude of the measurement error in observed measurements to the inherent variability in the “error-free” or “true” level of the quantity between subjects and is defined by $\frac{{\left(\mathrm{S}\mathrm{D}\ \mathrm{subjects}'\ \mathrm{true}\ \mathrm{values}\right)}^2}{{\left(\mathrm{S}\mathrm{D}\ \mathrm{subjects}'\ \mathrm{true}\ \mathrm{values}\right)}^2+{\left(\mathrm{S}\mathrm{D}\ \mathrm{measurement}\ \mathrm{error}\right)}^2}=\frac{{\left(\mathrm{S}\mathrm{D}\ \mathrm{between}\ \mathrm{subjects}\right)}^2}{{\left(\mathrm{S}\mathrm{D}\ \mathrm{between}\ \mathrm{subjects}\right)}^2+{\left(\mathrm{S}\mathrm{D}\ \mathrm{within}\ \mathrm{subjects}\right)}^2}$ [33, 34].

Reliability for single measurements of CFVR with CI was estimated from the reproducibility study as the intra-class correlation coefficient (ICC) from a one-way random-effects model.

For graphical representation of the correspondence between the CFVR and MFR, in addition to the Bland-Altman plot we used scatter plot with illustration of the reduced major axis regression line, a useful summary of data, defined as the line going through the intersection of the means with a slope given by the sign of the Pearson’s correlation *r* and the ratio of the respective standard deviations [35–37].

All analyses were performed in STATA/IC 13.1 (StataCorp LP, College Station, TX, USA).

## Results

### Feasibility of CFVR

Main participant characteristics, outlined in Table 1, did not differ significantly between participants and non-participants of the reproducibility and PET sub-studies. Median age was 63 years and median BMI was 30.9 kg × m^−2^.

**Table 1:** Patient characteristics

	Feasibility (*n* = 86)	Reproducibility (*n* = 21)	Method agreement (*n* = 35)
Male gender	72 (84 %)	20 (95 %)	32 (91 %)
Age [years]	63 (57–67)	63 (58–67)	63 (60–69)
Height [m]	1.74 (1.69–1.78)	1.75 (1.72–1.82)	1.77 (1.70–1.77)
Weight [kg]	92.8 (85.6–100.5)	90.6 (82.1–97.4)	92.8 (85.4–101.0)
BMI [kg × m^−2^]	30.9 (29.4–32.9)	29.0 (26.3–32.1)	30.8 (28.9–32.1)
MI	51 (59 %)	12 (57 %)	18 (51 %)
MI_LAD_	26 (30 %)	8 (38 %)	12 (34 %)
CABG	18 (21 %)	8 (38 %)	9 (26 %)
Atrial fibrillation	7 (8 %)	2 (10 %)	2 (6 %)
Use of contrast	12 (14 %)	1 (5 %)	8 (23 %)
CFVR	2.29 (1.90–2.63)	2.27 (1.82–2.69)	2.39 (1.89–3.04)

Categorical and continuous variables expressed as count (%) and median (IQR), respectively. *BMI* body mass index, *MI* myocardial infarction, *LAD* left anterior descending artery territory, *CABG* coronary artery bypass graft, *CFVR* coronary flow reserve by transthoracic Doppler echocardiography. No significant differences between participants and non- participants in reproducibility and PET study. *P* = 0.061(Fisher’s exact) for CABG in reproducibility study. *P* = 0.057 (Fisher”s exact) for contrast usage in PET study

CFVR showed high feasibility with successful measurement in 83 (97 %) of 86 participants, a contrast agent was applied in 12 (14 %). Median CFVR was 2.29, ranging from 1.33 to 3.95. Sixty-two (75 %) participants were examined with dipyridamole and 21 (25 %) with adenosine. Average heart rate increased by 18 beats per minute (*P* < 0.0001) during hyperaemia whereas systolic and diastolic blood pressure did not change significantly. No significant difference was observed between dipyridamole and adenosine in mean CFVR and response of heart rate or blood pressure. Median resting RPP was 8016 (6950;9406) and did not correlate with CFVR (*P* = 0.88).

### Reproducibility of CFVR

**Fig. 4:**
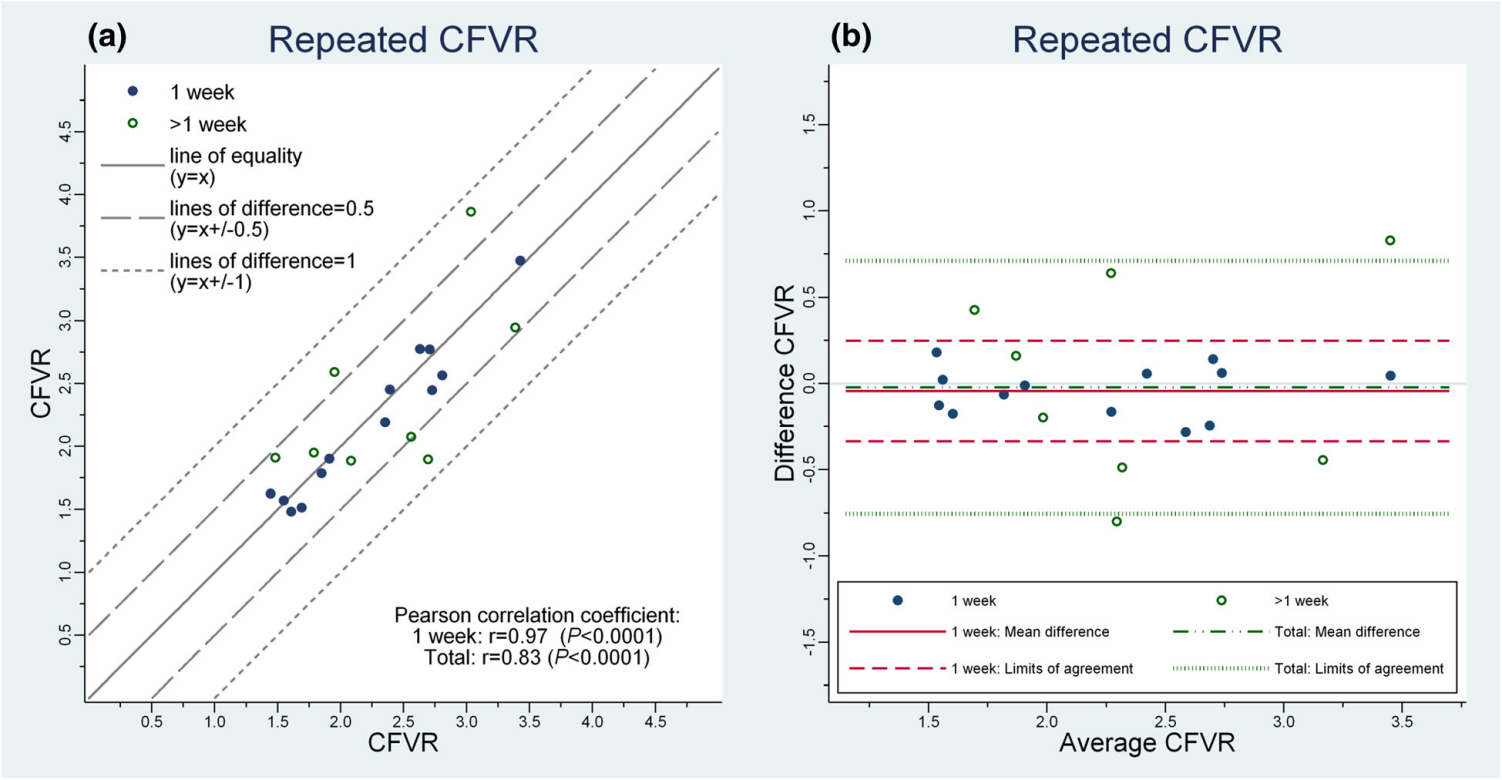
Reproducibility of CVFR (*n* = 21). **a** Scatter plot of repeated measurements of CFVR (coronary flow reserve by transthoracic Doppler echocardiography) performed in 1 week (*blue closed circles*) and more than a week apart (*green open circles*). Grey lines: full line marks equality (no difference), dashed lines mark absolute differences of 0.5, and dotted lines represent absolute differences of 1 between repeated exams. **b** Bland-Altman plot: Differences *vs* averages of repeated CFVR measurements performed in 1 week (*blue closed circles*) and more than a week apart (*green open circles*). Limits of agreement for exams performed within a week (*red dashed lines*) and combined for all exams (*green dotted lines*) are displayed together with corresponding mean differences (bias) for a week (*red full line*) and all differences (*green dashed and dotted line*)

CFVR of the reproducibility study are shown in Fig. 4a-b as scatter plot and difference *vs* mean (“Bland-Altman”) plot. Measurement error was uniform over the range of CFVR. Measures of reproducibility are summarized in Table 2.

**Table 2:** Reproducibility of repeated CFVR

	1 week (*n* = 13)	>1 week (*n* = 8)	Total (*n* = 21)
CFVR Mean	2.22 (0.59)	2.38 (0.60)	2.28 (0.59)
Mean difference	−0.044 (−0.13;0.044)	0.016 (−0.48;0.51)	−0.021 (−0.19;0.15)
2SD	0.29 (0.16;0.42)	1.17 (0.43;1.92)	0.73 (0.49;0.97)
CV repeated exams [%]	7 (4;10)	25 (9;40)	16 (11;21)
CV repeated exams RPP corrected [%]	12 (7;17)	17 (6;28)	14 (9;18)
CV of RPP [%]	12 (7;17)	18 (7;29)	14 (9;19)
Reliability, ICC	0.97 (0.91;0.99)	0.67 (0.05;0.92)	0.84 (0.65;0.93)
SD between-subjects	0.58 (0.39;0.85)	0.51 (0.27;0.97)	0.56 (0.40;0.78)
SD within-subjects	0.10 (0.07;0.15)	0.39 (0.24;0.63)	0.25 (0.19;0.34)
CV within-subjects [%]	5 (3;7)	16 (10;27)	11 (8;15)

Estimates of reproducibility for CFVR (coronary flow velocity reserve measured by transthoracic Doppler echocardiography) for repeated measurement within 1 week, more than 1 week, and for the total population of the sub-study. CFVR is mean (SD), all other variables are estimate (CI). *2SD* Two times standard deviation of differences, *CV* coefficient of variation, *ICC* intra-class correlation coefficient, *RPP* rate-pressure product, *SD* standard deviation

The reproducibility of CFVR was overall acceptable with a reliability of 0.84 (corresponding Pearson correlation coefficient *r* = 0.83, *P* < 0.0001), (−0.75;0.71) and within-subject CV of 11 %. Exams repeated within a week showed better reproducibility with reliability of 0.97 (*r* = 0.97, *P* < 0.0001), LOA (−0.33;0.25) and within-subject CV of 5 %, which was significantly better than for exams performed longer apart (*P* = 0.0001 for equal SD of differences). The within-subject CV(CI) of CFV during rest and hyperaemia was 15(11;26) % and 17(12;23) % respectively.

In terms of characteristics listed in Table 1 patients examined within a week did not differ from patients with examinations performed longer apart. Reproducibility did not differ between patients with and without prior MI (all: *P* = 0.44 and LAD-territory: *P* = 0.51) nor between examinations using adenosine and dipyridamole (*P* = 0.50, *n:* 9 vs. 12).

CFR may be affected by baseline oxygen consumption. However, difference in CFVR and RPP between repeated exams did not correlate (*P =* 0.24) and correction of CFVR for resting RPP did not improve reproducibility in terms of CV (*P* = 0.20).

### Method agreement: CFVR vs. MFR

**Fig. 5:**
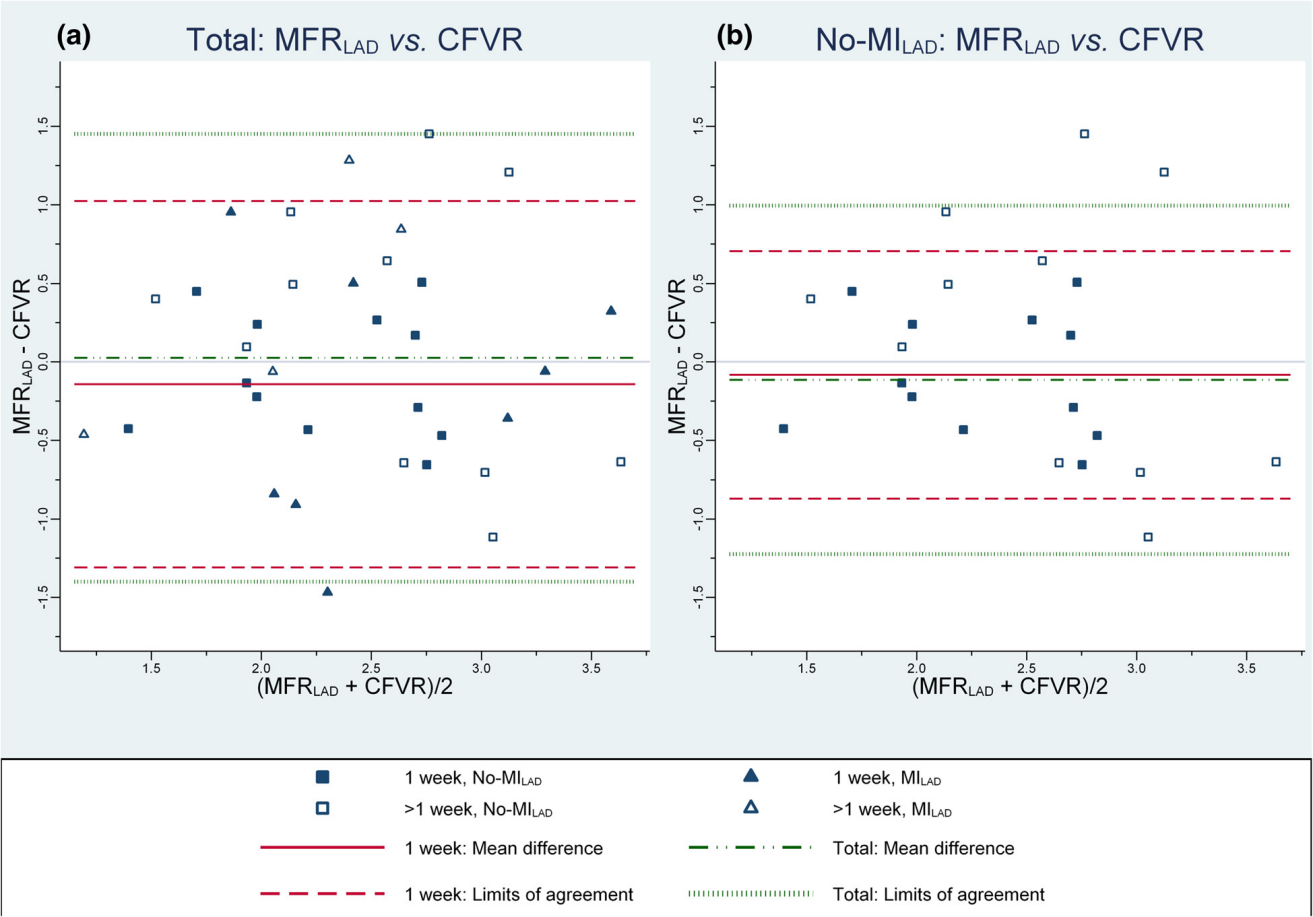
Method agreement CFVR vs MFR_LAD_. “Bland-Altman plot”: Differences *vs* averages of myocardial flow reserve (MFR) measured by PET of the LAD-territory (MFR_LAD_) versus coronary flow velocity reserve (CFVR) measured by transthoracic Doppler echocardiography of the LAD. **a** include all participants and (**b**) display measurements of participants with no prior myocardial infarction (No-MI) of the LAD territory. Examinations performed in a week are marked by closed squares and triangles for participants without and with prior myocardial infarction of the LAD territory, respectively. Examinations performed more than a week apart are marked by open squares and triangles for participants without and with prior myocardial infarction of the LAD territory, respectively. Limits of agreement for exams performed within a week (*red dashed lines*) and combined for all exams (*green dotted lines*) are displayed together with corresponding mean differences (bias) for a week (*red full line*) and all differences (*green dashed and dotted line*)

**Fig. 6:**
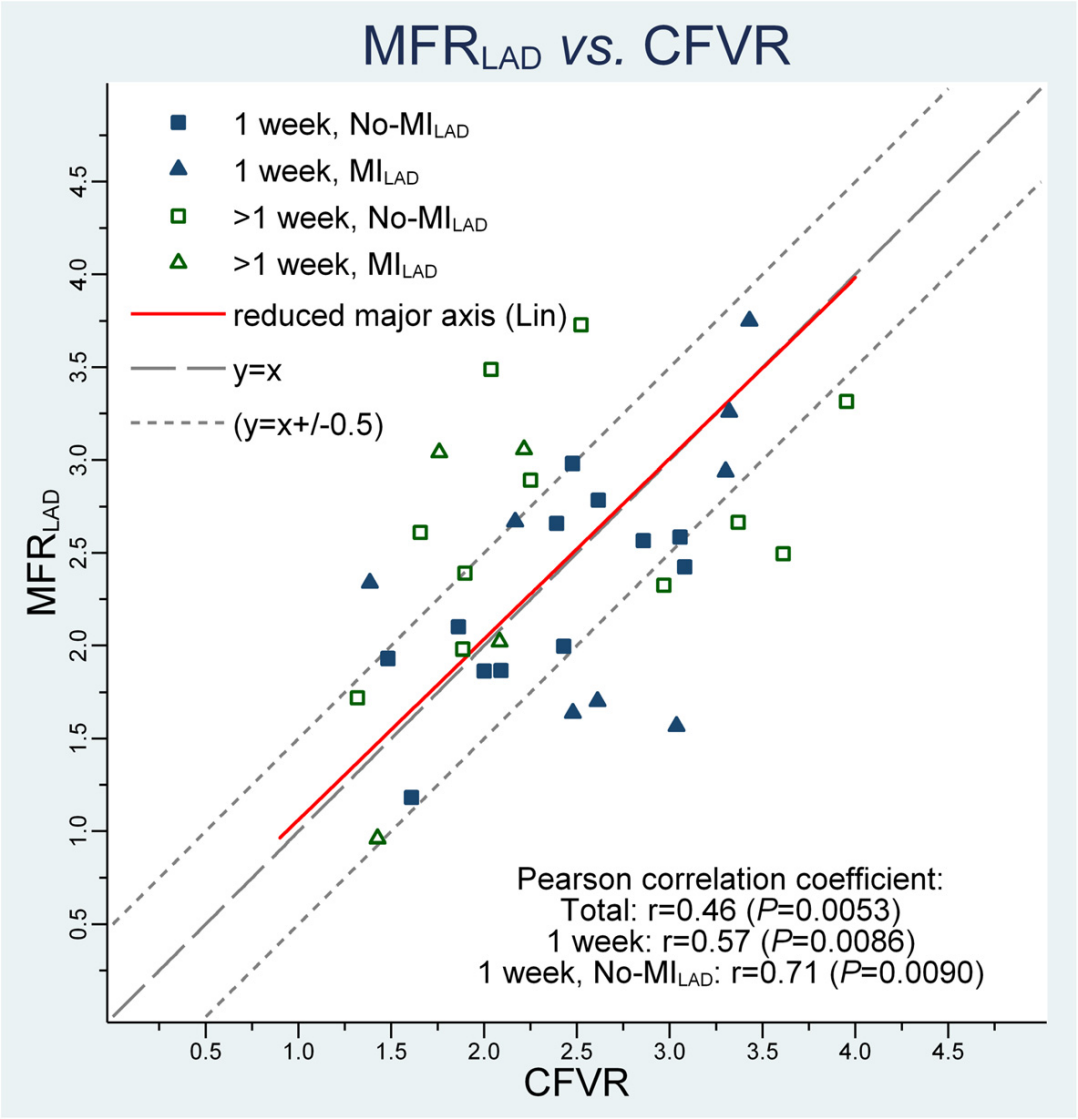
Method agreement MFR_LAD_ CFVR vs CFVR. Scatter plot of myocardial flow reserve (MFR) measured by PET of the LAD-territory (MFR_LAD_) versus coronary flow velocity reserve (CFVR) measured by transthoracic Doppler echocardiography of the LAD. Examinations performed in a week are marked by blue closed squares and triangles for participants without and with prior myocardial infarction of the LAD territory, respectively. Examinations performed more than a week apart are marked by green open squares and triangles for participants without and with prior myocardial infarction of the LAD territory, respectively. Full red line: represents the reduced major axis, the line going through the intersection of the means with a slope given by the sign of the Pearson’s correlation *r* and the ratio of the respective standard deviations. Grey lines: dashed line marks equality (no difference), dotted lines mark absolute differences of 0.5 between measurements

Thirty-five patients participated in the method agreement study. Overall, MFR_global_ was 2.46(0.61), MFR_LAD_ 2.44(0.68) and CFVR 2.42(0.70). The means were not different between participants with and without MI (*P* = 0.24, 0.33 and 0.84). Agreement of CFVR with MFR_LAD_ is summarized in Table 3 and Fig. 5. The correlation between CFVR and MFR_LAD_ was modest but significant (*r* = 0.46, *P* = 0.0053) and with no systematic bias (Fig. 6). Agreement tended to be better for examinations performed within a week (1-week vs. >1-week: *P* = 0.059 for equal SD of differences). In terms of characteristics listed in Table 1, patients examined within a week did not differ from patients with examinations performed longer apart. Exclusion of participants with prior MI improved correlation and agreement with PET (test of equal SD of differences between CFVR and MFR_LAD_ of No-MI_LAD_ vs. MI_LAD_: *P* = 0.024 for exams within a week and *P* = 0.38 totally). The agreement between methods was best for participants without MI with exams performed within a week (for No-MI_LAD_: 1-week vs. >1-week: *P* = 0.0072) with LOA = (−0.68;0,88) corresponding to CV = 17 % for MFR_LAD_. Agreement with MFR_global_ was as least as good; overall 2SD (CV) was 1.32 (27 %), for scans performed within a week 1.05 (22 %), and for scans within a week in participants without prior MI of any perfusion territory 0.49 (11 %).

**Table 3:** Agreement between CFVR measured by echocardiography and MFR measured by PET of the LAD territory (*n* = 35)

	1 week			Total		
MFR_LAD_ – CFVR	No-MI_LAD_ (*n* = 12)	MI_LAD_ (*n* = 8)	Combined (*n* = 20)	No-MI_LAD_ (*n* = 23)	MI_LAD_ (*n* = 12)	Combined (*n* = 35)
Pearson correlation, *r*	0.71 (0.0090)	0.43 (0.29)	0.57 (0.0086)	0.49 (0.018)	0.44 (0.16)	0.46 (0.0053)
Mean difference	0.11 (−0.33;0.17)	−0.23 (−0.91;0.45)	−0.14 (−0.42;0.13)	0.05 (−0.40;0.17)	−0.02 (−0.55;0.53)	0.03 (−0.22;0.27)
2SD	0.79 (0.42;1.16)	1.63 (0.60;2.66)	1.17 (0.77;1.56)	1.32 (0.91;1.73)	1.67 (0.89;2.45)	1.43 (1.07;1.78)
CV [%]	17 (9;25)	31 (12;51)	24 (16;32)	27 (19;36)	34 (18;51)	29 (22;37)

Values are given as estimate (*P* or CI). *CFVR* Coronary flow velocity reserve by echocardiography of the LAD, *MFR*_*LAD*_ Myocardial flow reserve of the LAD territory by PET, *MI*_*LAD*_ Myocardial infarction of the LAD territory, *2SD* Two times standard deviation of differences, *CV* coefficient of variation

Agreement was not different between patients with prior MI of the LAD-territory and MI of other territories (MFR_LAD_: *P* = 0.59), nor was it affected by CABG-status (MFR_LAD_: *P* = 0.78), or time between revascularization and examination (MFR_LAD_: *P* = 0.91), which was median(range) 2(0,5-16) years. There was no difference in mean subject SD of MFR with any of the strata MI or time between examinations.

Resting RPP correlated with PET estimated resting perfusion (LAD: *r* = 0.57, *P* = 0.0007), but not with MFR (MFR_LAD_: *P* = 0.14). Correction for resting RPP did not improve agreement between methods nor did differences in resting RPP and differences between MFR and CFVR correlate (MFR_LAD_: *P* = 0.61).

## Discussion

The present study shows high feasibility of CFVR in a population with generally limited echocardiographic acoustic window. Reproducibility of CFVR was good especially when performed within a week. Agreement with PET was overall modest, primarily because of poor agreement between methods in participants with prior MI whereas agreement with PET was good in participants without prior MI. Both the reproducibility and method agreement sub-studies indicate that variability over time in CFR should be taken into account e.g. when planning studies with purpose of testing the effect of interventions on CFR.

### Feasibility of CFVR

Although CFVR has low cost compared to other CFR methods and advances in echocardiographic technology have led to improvement in accessibility, CFVR has not achieved wide routine use in clinical practice, primarily due to concerns of feasibility in representative patient populations and validity of the measures achieved [38].

We found a feasibility of 97 % in an unselected group of patients with a median BMI above 30 kg × m^−2^. This feasibility is comparable to those previously obtained in patient populations not excluding overweight subjects. A large multicentre study comprising 1544 patients had a feasibility of 92 % (exclusive of 2 % for whom examination was stopped prematurely because of side-effects) with contrast used in 36 % [7]. One study with a population mean BMI of 30 (*n* = 38) reported feasibility of 92 % with 10 % requiring contrast usage and other studies not reporting on body habitus have achieved success rates up to 100 % (*n* = 124) [9, 39]. In comparison feasibility of 95 and 98 % has been reported for mitral annular velocities as measured by speckle tracking and PW tissue Doppler, respectively [40].

### Reproducibility of CFVR

LOA of CFVR repeated within a week in the present study is almost identical to LOA of 0.32 in a study of 13 patients referred for CAG in whom CFVR was repeated with a delay of one hour, and to LOA of 10 % in hypertensive patients (*n* = 8) examined 3–5 days apart, as well as a CV of 6 % reported for lean healthy subjects (*n* = 8) [12, 18, 41]. In fact the reproducibility could not be expected to be better, since the present within-subject CV, corresponding to error of measurement, is on par with the previously reported observer variability of repeated off-line readings [24]. The within-subject CV tends to be higher for CFV than CFVR, which probably is due to the fact that minor differences between examinations that may cause difference in measured CFV between examinations (e.g. placement of probe or sample volume and Doppler angle to the flow), tends to have no significant impact on CFVR as long as it is kept constant during the same examination.

CFVR seems to perform as well as several other measures applied in clinical practice. The reproducibility of CFVR repeated within a week is comparable to measures as LVEF by Simpson biplane method, M-Mode mitral annular excursion, and peak early mitral annular velocity (e’) (CV 5–12 %) in terms of CV [40, 42, 43]. Similarly, we find the overall reproducibility of CFVR to be at least as good as for MFR by PET (CV range 17 to 26 %) [30, 44–46].

The good reproducibility of CFVR has practical implications as it underpins the usefulness of serial evaluation of CFVR e.g. before and after revascularization, or as an outcome measure in clinical as well as preclinical trials of e.g. drug therapy on coronary microvascular function.

Reliability is an estimate of the proportion of all variation that is not due to measurement error [33, 34]. For laboratory measurements a reliability above 0.90 is desirable [34]. We report reliability for examinations repeated within a week that is significantly higher, meaning that more than 90 % of the variability in the measurements of CFVR was due to genuine differences in CFVR between the participants. Reliability is influenced by the heterogeneity (variance) of the study population with regard to the measured parameter, i.e. the greater between-subjects variance the greater reliability. As we have made no selection on CFVR values, the reported reliability is representative for a population of overweight and obese revascularized and stable CAD patients. The relatively high reliability of CFVR makes it suited for distinguishing patients on this parameter. This is also reflected in its ability to prognosticate [6].

### Method agreement: CFVR vs. MFR

There are obvious differences between methods of estimating CFVR by TTE Doppler and MFR by PET. CFVR is the ratio of peak diastolic flow velocities whereas MFR estimates the perfusion volume-velocity per myocardial tissue mass (mL × g^−1^ × min^−1^) for the entire heart cycle. Further, the methods can be affected be different factors. For example, a change between rest and hyperaemia in the calibre of the artery where the sample volume is placed would have effect on the flow velocity and the measured CFVR. Whereas, ^82^Rb extraction can be decreased by severe acidosis, hypoxia, and ischemia; thus in addition to blood flow, ^82^Rb uptake could be affected by metabolism and myocardial cell integrity [47].

We found 2SD ranging from 0.49 to 1.67. For comparison, we have knowledge of only one study; Saraste et al. examined CFVR and MFR_LAD_ in 10 young, healthy participants with a mean delay of 13 days, and reported LOA corresponding to mean difference (2SD) of −0.20 (1.03) [18]. Thus, agreement for obese CAD patients performed within a week, −0.14(1.17), is of comparable magnitude to that of the healthy, lean and young subjects.

Agreement between CFVR and MFR_global_ was as good as for CFVR and MFR_LAD_. Possible explanations are that microvascular dysfunction is a process that affects the global myocardium and the reproducibility of regional estimates tend to be poorer than for global estimates in the majority of studies [30, 44–46, 48].

In concordance with our findings for agreement between CFVR and MFR, reproducibility of MFR is in general not improved by correction for RPP, nor is it the tradition to correct CFVR for RPP [30, 44–46, 48].

Agreement between any two methods depends on and is limited by the repeatability or reproducibility of both methods [31]. We did not evaluate the reproducibility of MFR but it has been assessed previously by others. Reproducibility seems to be similar for ^15^O-water, ^13^N-ammonia or ^82^Rb, and without any obvious difference between studies of delayed or immediately repeated exams [30, 44–46, 48, 49]. Sdringola et al. evaluated the reproducibility of MFR using ^82^Rb in 107 healthy subjects with a median delay of 22 days between repeated exams. For global estimates (regional being of comparable magnitude) they found reproducibility corresponding to LOA +/−38 % and +/−51 % of mean MFR, equivalent to absolute differences of 1.6 and 2.0 in what they termed “true” and “not-true normals”, respectively [44]. Manabe et al. applied ^82^Rb in repeated exams separated by an hour and found reproducibility corresponding to LOA of +/−1.6 (37 %) in15 healthy participants [46].

In this context, our overall findings of agreement between CFVR and MFR_LAD_ and MFR_global_ must be considered to be acceptable.

The acceptable agreement between CFVR and MFR can have practical implications in the clinical setting as CFVR as a non-invasive, non-ionising method with prompt availability and lower cost would be preferable.

Our data of both CFVR reproducibility and method agreement indicate that variability over time in CFR should be taken into account e.g. when planning studies with purpose of testing the effect of interventions on CFR. That CFR and microvascular function is a dynamic parameter is also illustrated by previous findings of improvement in CFR early (days) after stenting both in patients with and without MI, with further improvement in some but not all patients after 3–6 months [50–52], which can relate to myocardial recovery [52], and in-stent restenosis [9, 51]. Likewise, improvement in CFR has been illustrated from 1 to 6 months after CABG [53]. The post interventional improvement could also be influenced by rehabilitation efforts such as exercise and weight-loss [8]. In attempt to reduce the potential effect of recovery after MI, PCI, in-stent restenosis and CABG we included only participants in stable phase at least six months after PCI, MI or CABG.

### Limitations

We instructed participants and they agreed to abstain from caffeine before examinations, but we did not asses the validity of this by means of laboratory testing of caffeine levels as it would normally not be done in a clinical setting. If any participant should not have complied with the instructions on caffeine, it must be considered a random effect and as so it would tend to decrease the estimated reproducibility and agreement. It is a limitation of the method agreement substudy that only patients with revascularized stenosis were included, however the focus of the present study was on CFR as a measure of microvascular function and not on estimation of stenosis severity. We did not repeat assessment of coronary anatomy immediately before trial start. However, exercise ecg and stress echocardiography were performed at inclusion and these were without limiting angina or signs of ischemia in all patients included in the study. This was also illustrated by the fact that CFVR of LAD had as good agreement with MFR_global_ as with MFR_LAD_.

CFVR was measured as distal as possible, in order to measure distal to grafts to the LAD. Accordingly, we consider CFVR a measure of microvascular function also in the participants with CABG.

Patients with previous CABG often have complicated coronary anatomy and thus, it can be difficult to define “matched” myocardial region perfused by the LAD vs. competitive flow from grafts, LCX or RCA for comparisons with CFVR [54]. We did not take into account the exact coronary anatomy from CAG of the individual participant, and the regional MFR was estimated by our clinical routine practice from the standard region applied by the software. However, our agreement obtained in non-MI participants within a week is not far from agreement between repeated readings (the same examination) of MFR performed with different software-packages (LOA +/−0.3, *n =* 90 patients) or between a novice and an expert reader using identical software (LOA +/−0.3, *n =* 30) [55, 56].

The sample size was limited and it was not the purpose of the study to estimate differences in agreement between subgroups (ie MI vs no-MI, and PCI vs CABG).

## Conclusions

In conclusion, CFVR had high feasibility with the potential use of a contrast agent. Reproducibility was good compared to previous estimates for lean and healthy subjects, and compared to reproducibility of other well-known measures used in the field of cardiology. Acceptable agreement with PET MFR was achieved, though discrepancy related to prior MI has to be considered and evaluated in future studies. Our study thus confirms that CFVR by TTE is a feasible and valid method for assessing microvascular function in stable and revascularized overweight and obese patients with CAD.

## Abbreviations

2SD, two times the standard deviation of the differences; ^82^Rb, rubidium-82; BMI, Body mass index; CABG, coronary artery bypass graft; CAD, coronary artery disease; CFR, coronary flow reserve; CFV, coronary flow velocity; CFVR, coronary flow velocity reserve; CI, confidence interval; CV, coefficient of variation; ICC, intra-class correlation coefficient; IQR, interquartile range; LAD, left anterior descending coronary artery; LOA, limits of agreement; MBF, myocardial blood flow; MFR, myocardial flow reserve; MI, myocardial infarction; PCI, percutaneous coronary intervention; PET, positron emission tomography; RPP, rate-pressure product; SD, standard deviation; TTE, transthoracic echocardiography
